# From Single‐Antigen Beads to the Physical Crossmatch: A Class‐Specific MFI Framework for Virtual Crossmatching in Kidney Transplantation

**DOI:** 10.1002/jcla.70400

**Published:** 2026-11-13

**Authors:** Nuchjarnun Choktaweesak, Supasiri Ounjai, Patcharipa Chanaturakarnnon, Nattiya Hirankarn, Suwasin Udomkarnjananun

**Affiliations:** ^1^ Histocompatibility and Immunogenetics Laboratory National Blood Center, Thai Red Cross Society Bangkok Thailand; ^2^ Medical Sciences Program, Graduate Affairs, Faculty of Medicine Chulalongkorn University Bangkok Thailand; ^3^ Department of Microbiology, Faculty of Medicine, Center of Excellence in Immunology and Immune‐Mediated Diseases Chulalongkorn University Bangkok Thailand; ^4^ Division of Nephrology, Department of Medicine, Faculty of Medicine King Chulalongkorn Memorial Hospital and Chulalongkorn University Bangkok Thailand; ^5^ Excellence Center for Solid Organ Transplantation King Chulalongkorn Memorial Hospital, Thai Red Cross Society Bangkok Thailand; ^6^ Center of Excellence Renal Immunology and Renal Transplantation, Faculty of Medicine Chulalongkorn University Bangkok Thailand

**Keywords:** complement‐dependent cytotoxicity crossmatch, donor‐specific antibody, flow cytometry crossmatch, HLA, kidney transplantation, mean fluorescence intensity, single‐antigen bead, virtual crossmatch

## Abstract

**Background:**

As kidney transplantation shifts toward virtual crossmatching using single‐antigen bead (SAB) mean fluorescence intensity (MFI), understanding how donor‐specific antibody (DSA) thresholds align with physical crossmatches is critical. This study mapped class‐specific MFI thresholds, testing whether SAB, flow cytometric (FCXM), and complement‐dependent cytotoxicity (CDC) crossmatches form an ordered continuum.

**Methods:**

We retrospectively analysed 103 sensitized, SAB‐DSA‐positive candidates with paired CDC crossmatches (52 with additional FCXM). Maximum DSA MFI per HLA class was correlated with crossmatch results. Discrimination was assessed via AUC. The B‐cell crossmatch was analysed by DSA profile (isolated Class I, isolated Class II, coexisting) with bivariable regression and a jointly optimized Class I/Class II rule.

**Results:**

SAB, FCXM, and CDC formed a sensitivity hierarchy (SAB > FCXM > CDC) accompanied by rising MFI (*p* < 0.001). No CDC‐positive candidate was flow‐negative. Maximum Class I MFI effectively discriminated the T‐cell crossmatch (CDC AUC 0.94, FCXM AUC 0.89), while Class II MFI did not (AUC 0.50 in coexisting DSA). When both classes were present, Class I and Class II each contributed independently to the CDC B‐cell crossmatch (adjusted OR 6.65 and 7.34 per 10‐fold MFI). A jointly optimized rule (Class I ≥ 16,482 or Class II ≥ 7928) predicted a positive CDC B‐cell crossmatch with 100% sensitivity and 100% negative predictive value in every DSA profile.

**Conclusions:**

SAB, FCXM, and CDC crossmatching form a biologically ordered continuum. Safe virtual crossmatching requires class‐ and assay‐specific MFI thresholds. Beyond predicting compatibility, these cut‐offs may guide individualized immunosuppression and define quantitative targets for desensitization protocols.

## Graphical abstract

Single‐antigen bead (SAB) assays, flow cytometric (FCXM), and complement‐dependent cytotoxicity (CDC) crossmatches form an ordered continuum of analytic sensitivity accompanied by rising donor‐specific antibody (DSA) strength. To predict B‐cell crossmatch positivity, this study provides both a single overall maximum MFI threshold and a jointly optimized “either/or” rule based on distinct HLA Class I and Class II cut‐offs. Interpreting SAB reports through this biologically validated framework offers safer, class‐specific guidance for virtual crossmatching.

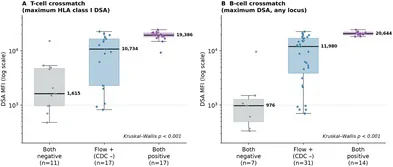

## 1 Introduction

Since Patel and Terasaki showed that a positive complement‐dependent cytotoxicity (CDC) crossmatch predicts hyperacute rejection, the physical crossmatch has been the final gate before kidney transplantation [1]. CDC detects only antibody dense enough, and sufficiently complement‐fixing, to lyse donor lymphocytes, and its role as gold standard carries a recognized ceiling of sensitivity [2]. The flow cytometric crossmatch (FCXM) captures lower‐density binding that CDC misses [3], and solid‐phase single‐antigen bead (SAB) assays resolve anti‐HLA antibody to individual specificities, reporting strength as a semiquantitative mean fluorescence intensity (MFI) [4]. The three assays thus probe the same alloimmune response at progressively lower thresholds of antibody quantity.

Sensitized candidates face longer waiting times and reduced access to organs [5], and preformed donor‐specific antibody (DSA) is associated with antibody‐mediated rejection and inferior graft survival [6]. To widen access, programs increasingly use the virtual crossmatch, comparing the SAB‐defined DSA profile against the donor HLA type to predict compatibility before—or instead of—a physical crossmatch [7]. This shortens cold ischemia and avoids unnecessary crossmatches, but rests on an assumption the older paradigm never had to quantify: that a given DSA MFI reliably corresponds to a given physical crossmatch result. Because MFI is semiquantitative and vulnerable to the prozone effect, that correspondence must be established empirically [8].

Several groups have reported MFI cut‐offs for crossmatch positivity, but the evidence is fragmented—single HLA class or lineage, differing protocols, and a single global threshold [9, 10, 11, 12, 13, 14]. Two problems recur. The B‐cell crossmatch reflects both HLA classes, so a positive result cannot be assigned to one class when both are present—leading some to exclude it from MFI‐correlation analyses [11]. Others have restricted the Class II analysis of the B‐cell crossmatch to Class I‐negative candidates to minimise confounding by Class I antibody [15]. Thresholds derived on different platforms, using immunodominant versus cumulative MFI, are not directly comparable, leaving the field without a coherent, class‐resolved map of where each crossmatch turns positive.

The present study addresses that gap in 103 uniformly SAB‐DSA‐positive candidates with paired CDC and flow crossmatches. Rather than seeking another cut‐off, it asks whether CDC, FCXM and SAB form a single sensitivity continuum governed by rising DSA MFI, whether flow positivity strictly contains CDC positivity, and whether these relationships follow HLA‐class biology—T lymphocytes expressing only Class I, B lymphocytes both—closely enough to yield class‐specific decision rules, using the Class II signal for the T‐cell crossmatch as a built‐in biological control and analyzing the B‐cell crossmatch within a combined Class I/Class II framework stratified by DSA profile. The aim is a quantitative, test‐ and class‐specific basis for reading a SAB report and for judging when a physical crossmatch adds information beyond the virtual crossmatch.

## 2 Methods

### 2.1 Study Design and Population

This retrospective study is reported in accordance with the STROBE recommendations [16]. Histocompatibility testing performed at the Histocompatibility and Immunogenetics Laboratory, National Blood Centre, Thai Red Cross Society, between 2014 and 2020 was analysed. A total of 103 sensitized kidney transplant candidates were included, each with at least one DSA detected by single‐antigen bead testing and a complete CDC T‐ and B‐cell crossmatch; 52 additionally underwent flow cytometry crossmatch (FCXM). HLA antibodies were grouped into Class I (A, B) and Class II (DRB1, DRB3/4/5, DQB1). Antibody strength was summarised two ways: the immunodominant MFI—the single highest MFI within a class—as the primary measure, and the summed (cumulative) MFI—the sum of all MFI within that class—as a sensitivity analysis. Two class‐independent measures were also defined: the overall maximum—the single highest DSA MFI of either class, that is, the strongest antibody a candidate carried—and the summed Class I + II MFI—the total of all DSA MFI across both classes. HLA‐C and ‐DPB1 were not assessed. For the B‐cell analyses, candidates were classified by DSA profile as isolated Class I, isolated Class II, or coexisting Class I and Class II DSA.

### 2.2 Ethical Considerations

The study was approved by the Research Ethics Committee, National Blood Centre, Thai Red Cross Society (NBC 4/2569), and conducted in accordance with the Declaration of Helsinki. Because it used existing, de‐identified laboratory data, individual informed consent was waived. The study adhered to the Declaration of Istanbul on Organ Trafficking and Transplant Tourism; no organs were procured from executed prisoners or from any paid or coerced donor, and all donations were voluntary. All data were anonymized before analysis.

### 2.3 CDC Crossmatch

The CDC crossmatch was performed by the standard microcytotoxicity technique in accordance with American Society for Histocompatibility and Immunogenetics (ASHI) standards [17, 18]. Peripheral blood mononuclear cells were isolated by Ficoll density‐gradient centrifugation, and T and B lymphocytes separated on a nylon‐wool column. Donor lymphocytes were incubated with dithiothreitol (DTT)‐treated and untreated serum at 4, 22°C and 37°C. Patient serum was treated by mixing 20 μ L of 0.05 M DTT working solution with 180 μ L of serum (yielding a final DTT concentration of 5 mM) and incubating the mixture at 37°C for 45 minutes. Both DTT‐treated and untreated sera were subsequently diluted and tested in parallel. Cytotoxic reactions observed in untreated serum but abolished by DTT were attributed to IgM and recorded as negative, whereas reactions persisting after DTT treatment were considered IgG‐mediated and scored as positive [19]. Positive or negative status was determined by the more sensitive anti‐human globulin–augmented technique (CDC‐AHG). Cell death was read by inverted phase‐contrast microscopy after eosin and formaldehyde staining and graded 0–8; a score of 4 or greater (> 20% cell death) defined a positive crossmatch. This AHG‐augmented, IgG‐based assay is termed the CDC crossmatch throughout.

### 2.4 Flow Cytometry Crossmatch

The FCXM detected surface‐bound IgG on donor lymphocytes using a fluorescence‐labelled anti‐human IgG conjugate, so that only IgG was scored. Pronase‐treated and untreated lymphocytes were tested against patient serum, stained with a cocktail of FITC‐conjugated F(ab')_2_ goat anti‐human IgG (Jackson ImmunoResearch, West Grove, PA, USA) with anti‐CD3, ‐CD19, ‐CD20 and ‐CD45 (Beckman Coulter, Brea, CA, USA), fixed in 1% paraformaldehyde, and acquired on an FC500 cytometer within 24 hours. Positivity was defined by the median channel shift relative to the negative control, using the laboratory‐validated cut‐off of 50 channels or greater—within the range reported for cell‐based flow crossmatching [20].

### 2.5 HLA Antibody Identification and DSA Quantification

HLA antibodies were identified on a Luminex platform using LABScreen Single Antigen Beads, Class I and Class II (One Lambda, Canoga Park, CA, USA). 20 μL of neat serum was incubated with 5 μL of beads for 30 min at room temperature, washed, labelled with phycoerythrin‐conjugated anti‐human IgG and acquired on a LABScan 3D analyser with HLA Fusion software. Sera were not pre‐treated with EDTA, heat or DTT, and serial dilution to exclude a prozone effect was not routinely performed unless specifically requested. Raw (non‐baseline‐adjusted) MFI values were used for consistency with prior threshold studies, with an MFI of 500 or greater defining a positive bead [9, 10, 21, 22]. Donor specificity was assigned by matching antibody specificities to the donor HLA type, which was determined using intermediate‐resolution real‐time PCR for HLA‐A, ‐B, ‐DRB1, ‐DRB3/4/5 and ‐DQB1; DSA were therefore assigned at the antigen (serological‐equivalent) level, with allele‐level refinement only where typing allowed, and HLA‐C, ‐DQA1 and ‐DPA1/DPB1 were not typed. The immunodominant (maximum) MFI per class was the primary predictor, because a positive cell‐based crossmatch is a summed readout that cannot be attributed to a single specificity when several antibodies are present; the summed MFI per class was retained for the sensitivity analysis, and the summed Class I + II MFI was examined as a combined antibody‐load measure for the B‐cell crossmatch.

### 2.6 Statistical Analysis

Continuous MFI values were summarized as median (interquartile range [IQR]) and compared between crossmatch phenotypes with the Kruskal–Wallis test (across the ordered groups: both crossmatches negative; flow‐positive but CDC‐negative; both positive) and the Mann–Whitney *U* test. Categorical associations used the chi‐square or Fisher exact test with odds ratios and 95% confidence intervals (CI). CDC–flow concordance within each lineage was quantified with Cohen's κ, percent agreement and the McNemar test. Cohen's κ measures agreement beyond chance (by convention 0.21–0.40 fair, 0.41–0.60 moderate, 0.61–0.80 substantial) [23], and McNemar assesses whether discordant results fall predominantly in one direction; because κ is lowered by systematic discordance, attention was paid to the CDC‐positive/flow‐negative count as the marker of nested sensitivity. Maximum Class I and Class II MFI were correlated by the Spearman coefficient.

Discrimination of a positive crossmatch by MFI was quantified by the area under the receiver‐operating‐characteristic curve (AUC) with DeLong 95% CIs [24], for the CDC and flow crossmatch in each lineage. Optimal cut‐offs used the Youden index [25] with sensitivity and specificity, and operating characteristics were also tabulated at fixed thresholds of 3000, 5000, 10,000 and 15,000 MFI.

Because T lymphocytes express HLA Class I only, the T‐cell crossmatch was analysed against maximum Class I MFI, overall and separately in candidates with isolated Class I and with coexisting DSA, with Class II MFI in coexisting DSA as a pre‐specified biological negative control. Because B lymphocytes express both classes, a positive B‐cell crossmatch cannot be attributed to either class when both are present; therefore, the B‐cell crossmatch was analysed within a combined Class I/Class II framework. Class‐specific discrimination and cut‐offs were estimated only within the isolated Class I and isolated Class II subsets, where attribution is unambiguous. In coexisting DSA, the independent contribution of each class was tested by bivariable logistic regression (Class I and Class II log10 MFI) with likelihood‐ratio tests, odds ratios per 10‐fold MFI and a Class I × Class II interaction term, and combined measures (overall maximum and summed Class I + II MFI) were compared by paired DeLong tests. A combined “either/or” rule (positive if the strongest Class I or the strongest Class II DSA reached its threshold) was derived, separately for the CDC and the flow B‐cell crossmatch, by joint optimisation of both thresholds over all candidates tested, maximizing the Youden index across every pair of observed MFI values with an absent class counted as below threshold and reporting the lowest of any tied pairs; its optimism was estimated by re‐deriving the rule in 1000 bootstrap samples, and its performance was reported within each DSA profile. The same bivariable model applied to the T‐cell crossmatch served as a Class II specificity control. Because the dominant Class I effect caused separation in the T‐cell models, these used Firth penalized logistic regression, which gives finite, bias‐reduced estimates under separation, whereas the well‐identified CDC B‐cell model used standard maximum likelihood. Internal validity used 2000‐replication bootstrap CIs and optimism‐corrected AUCs, with a summed‐MFI sensitivity analysis. Locus‐level analyses were restricted to candidates with DSA at a single locus and are descriptive; antibody‐level specificity frequencies are exploratory because several antibodies from one candidate are not independent observations. Analyses used Stata 19 (StataCorp, College Station, TX, USA) and Python 3.12 (matplotlib); a two‐sided *p* < 0.05 defined significance.

## 3 Results

### 3.1 Study Population and DSA Characteristics

The analysis set comprised 103 sensitized candidates, all DSA‐positive by SAB and all with a complete CDC T‐ and B‐cell crossmatch; 52 additionally had a flow crossmatch (Table 1). The CDC crossmatch was positive in 19 candidates on the T‐cell (18.4%) and 22 on the B‐cell readout (21.4%). Among the 52 flow‐tested candidates the flow crossmatch was positive far more often—36/52 (69.2%) T‐cell and 45/52 (86.5%) B‐cell. Thirty candidates (29.1%) carried isolated Class I DSA, 23 (22.3%) isolated Class II DSA and 50 (48.5%) coexisting Class I and Class II DSA.

**TABLE 1:** Cohort and DSA characteristics.

Characteristic	Value
Candidates (all DSA‐positive by SAB)	103
With complete CDC T‐ and B‐cell crossmatch	103 (100%)
With flow crossmatch T and B	52
CDC T‐cell positive, *n* (%)	19 (18.4)
CDC B‐cell positive, *n* (%)	22 (21.4)
Flow T‐cell positive, *n* (%)	36/52 (69.2)
Flow B‐cell positive, *n* (%)	45/52 (86.5)
Max Class I DSA MFI, median (IQR)	6400 (2039–16,564)
Max Class II DSA MFI, median (IQR)	3398 (1137–12,357)
Max overall DSA MFI, median (IQR)	9539 (2124–17,865)
Isolated Class I DSA, *n* (%)	30 (29.1)
Isolated Class II DSA, *n* (%)	23 (22.3)
Coexisting Class I and Class II DSA, *n* (%)	50 (48.5)

Abbreviations: CDC, complement‐dependent cytotoxicity crossmatch; DSA, donor‐specific antibody; IQR, interquartile range; MFI, mean fluorescence intensity; SAB, single‐antigen bead.

### 3.2 A Rising DSA MFI Threshold Across the Test‐Sensitivity Hierarchy

When candidates were ordered by how demanding the positive test was—both crossmatches negative, flow‐positive but CDC‐negative, or both positive—the underlying DSA MFI rose monotonically (Figure 1). For the T‐cell crossmatch the median maximum Class I MFI rose from 1615 to 10,734 to 19,386 across these groups (Kruskal–Wallis *p* < 0.001), and for the B‐cell crossmatch the median maximum overall MFI rose from 976 to 11,980 to 20,644 (*p* < 0.001). CDC‐positive candidates carried stronger antibody than those positive only by flow (T‐cell *p* = 0.001; B‐cell *p* < 0.001), consistent with CDC requiring dense, complement‐fixing antibody.

**FIGURE 1:**
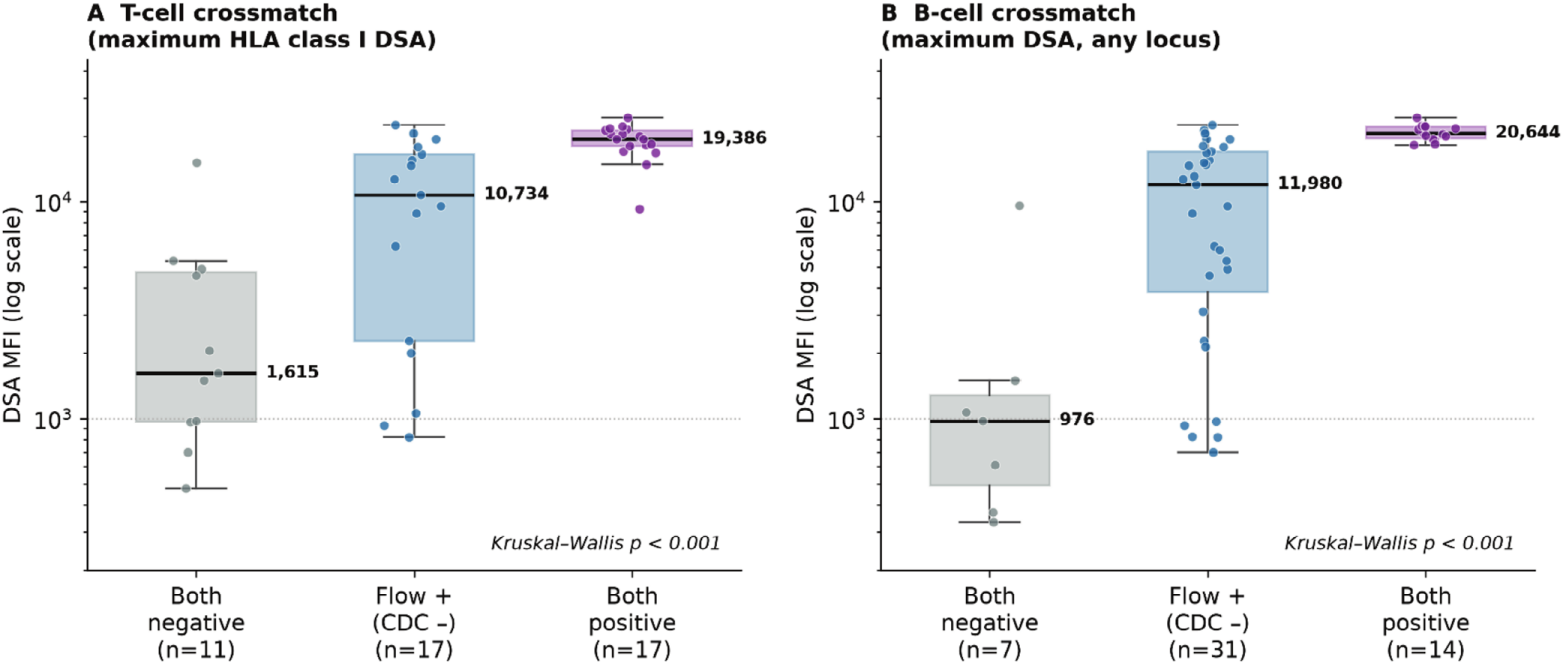
Rising DSA MFI threshold across the test‐sensitivity hierarchy (SAB → flow → CDC). Box‐and‐strip plots (log scale) of the maximum DSA MFI per candidate by crossmatch phenotype: (A) T‐cell, maximum Class I DSA; (B) B‐cell, maximum DSA at any locus. *p*‐values, Kruskal–Wallis.

### 3.3 Association Between Crossmatches and CDC‐Versus‐Flow Concordance

Within each method the T‐cell and B‐cell crossmatches were strongly associated (CDC *χ* ^2^ *p* < 0.001, *κ*: 0.54, 85% agreement; FCXM *χ* ^2^ *p* < 0.001, *κ*: 0.52, 83% agreement; Table 2, panels A and B). The decisive comparison is whether flow reproduces CDC (Table 2, panels C and D). In both lineages the CDC‐positive/FCXM‐negative cell was zero: every CDC‐positive crossmatch was also FCXM‐positive, while FCXM added 19 T‐cell and 31 B‐cell positives. The McNemar test was therefore highly significant and one‐directional, and Cohen's κ was only moderate (T‐cell 0.36; B‐cell 0.11) because the discordance was systematic rather than random—the signature of nested sensitivity.

**TABLE 2:** Association and concordance of the crossmatches (four panels).

Cross‐tabulation	Column−*n* (%)	Column+*n* (%)	Total
A. CDC: T‐cell (rows) × B‐cell (cols), *n* = 103 (*χ* ^2^ *p* < 0.001 *κ*: 0.54 agreement 85%)			
CDC T‐cell−	75 (89%)	9 (11%)	84
CDC T‐cell+	6 (32%)	13 (68%)	19
Total	81	22	103
B. Flow: T‐cell (rows) × B‐cell (cols), *n* = 52 (*χ* ^2^ *p* < 0.001 *κ*: 0.52 agreement 83%)			
Flow T‐cell−	7 (44%)	9 (56%)	16
Flow T‐cell+	0 (0%)	36 (100%)	36
Total	7	45	52
C. T‐cell: CDC (rows) × Flow (cols), *n* = 52 (McNemar *p* < 0.001 *κ*: 0.36 agreement 63%)			
CDC T‐cell−	16 (46%)	19 (54%)	35
CDC T‐cell+	0 (0%)	17 (100%)	17
Total	16	36	52
D. B‐cell: CDC (rows) × Flow (cols), *n* = 52 (McNemar *p* < 0.001 *κ*: 0.11 agreement 40%)			
CDC B‐cell−	7 (18%)	31 (82%)	38
CDC B‐cell+	0 (0%)	14 (100%)	14
Total	7	45	52

*Note:* Panels A and B test whether the T‐ and B‐cell crossmatches agree within a method (chi‐square); panels C and D test whether flow reproduces CDC (McNemar, paired). Cells are n (row %). In panels C and D the CDC‐positive/flow‐negative cell is zero, the signature of nested sensitivity: flow reproduced every CDC‐positive result and added 19 (T‐cell) and 31 (B‐cell) positives. *κ*, Cohen's kappa.

Within the CDC crossmatch, six candidates were T‐cell‐positive/B‐cell‐negative despite Class I DSA of 9234–21,349 MFI (Supplementary Table S1). Because B lymphocytes also express Class I, this pattern cannot be explained by antibody specificity; all six were positive in both lineages by flow, indicating a false‐negative B‐cell CDC reading—plausibly reflecting lower B‐cell viability after nylon‐wool separation and the higher background lysis against which B‐cell positivity is scored, a lineage‐threshold effect also described for the flow crossmatch [26]—rather than T‐cell‐restricted antibody.

### 3.4 T‐Cell Crossmatch and Class I DSA

Because T lymphocytes express only HLA Class I, the T‐cell crossmatch was analysed against Class I MFI (Figure 2A–B; Table 3; Supplementary Figure S1). Maximum Class I MFI discriminated the CDC T‐cell (AUC 0.94; 95% CI: 0.89–0.99) and flow T‐cell crossmatch (0.89; 0.79–0.99), with near‐identical CDC discrimination in isolated Class I and coexisting DSA (0.95 and 0.94). Within coexisting DSA, Class II MFI did not discriminate the CDC T‐cell crossmatch (AUC 0.50), the expected biological null. Its modest signal for the flow T‐cell crossmatch (0.69) disappeared after Firth‐penalized adjustment for Class I (Supplementary Table S2), and isolated Class II DSA never produced a positive CDC T‐cell crossmatch (0/23).

**FIGURE 2:**
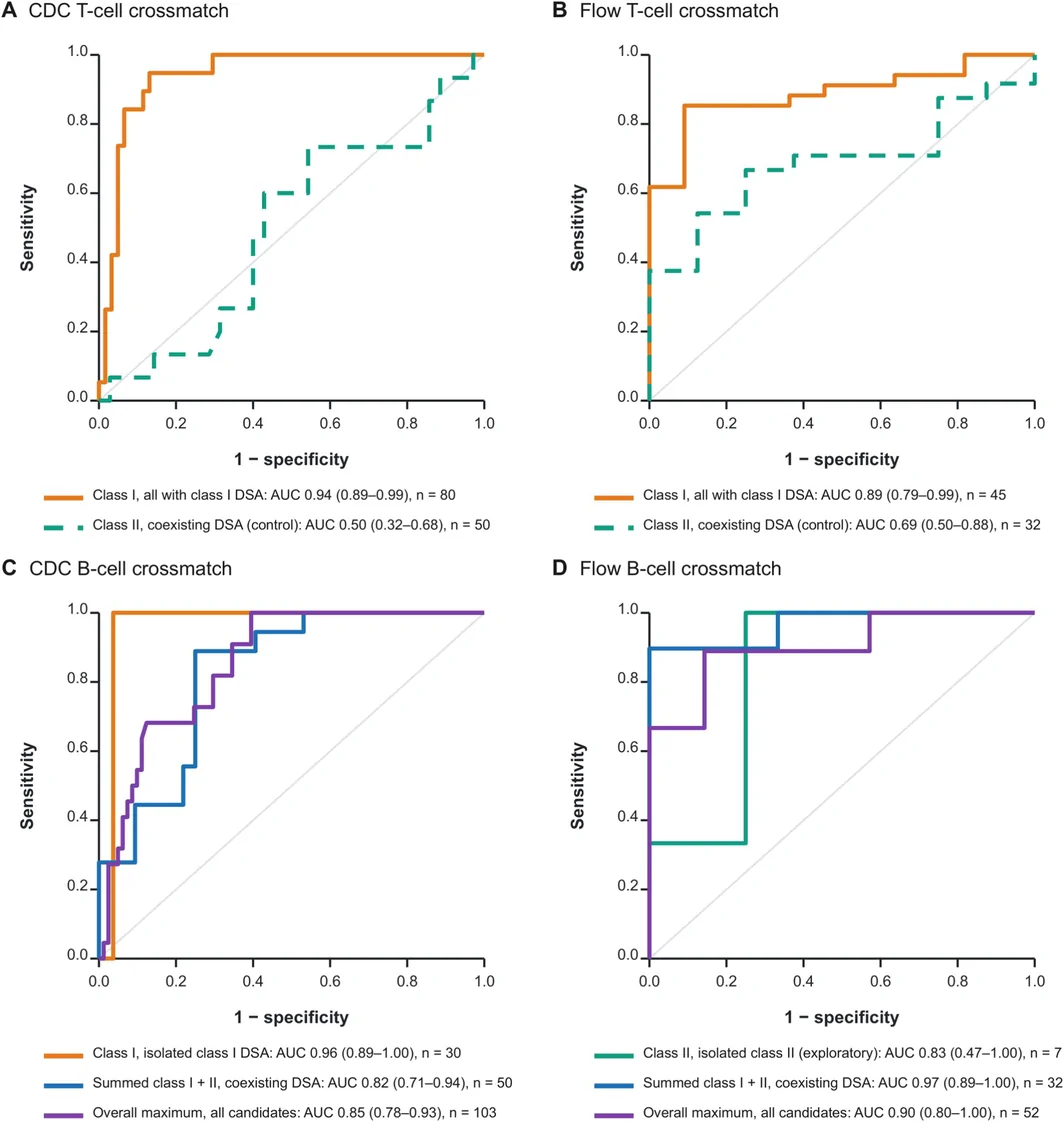
Receiver‐operating‐characteristic (ROC) curves of DSA MFI for each crossmatch. (A) CDC and (B) flow T‐cell crossmatch: maximum Class I MFI in all candidates with Class I DSA (orange) and maximum Class II MFI in candidates with coexisting DSA (teal, dashed), the biological negative control, as T cells lack Class II. (C) CDC and (D) flow B‐cell crossmatch within the combined Class I/Class II framework: Class I MFI in isolated Class I DSA (orange; CDC only, because every isolated Class I candidate was flow B‐cell‐positive), Class II MFI in isolated Class II DSA (teal; flow only and exploratory, *n* = 7, because only one isolated Class II candidate was CDC B‐cell‐positive), summed Class I + II MFI in coexisting DSA (blue), and overall maximum MFI in all candidates (purple). AUCs with 95% CIs (DeLong) are given beneath each panel. AUC, area under the curve.

**TABLE 3:** Discrimination of DSA MFI for the T‐cell crossmatch by Class I DSA (panel A) and for the B‐cell crossmatch within a combined Class I/Class II framework (panel B).

Crossmatch	DSA subset measure	*n* (pos/neg)	AUC (95% CI)	Youden cut‐off	Sens	Spec
A. T‐cell crossmatch (Class I DSA; T cells express Class I only)						
CDC T‐cell	All with Class I DSA Class I	80 (19/61)	0.94 (0.89–0.99)	14,239	95%	87%
CDC T‐cell	Isolated Class I	30 (4/26)	0.95 (0.87–1.00)	14,806	100%	92%
CDC T‐cell	Coexisting Class I	50 (15/35)	0.94 (0.88–1.00)	14,239	93%	86%
CDC T‐cell	Coexisting Class II (control)	50 (15/35)	0.50 (0.32–0.68)	—	—	—
Flow T‐cell	All with Class I DSA Class I	45 (34/11)	0.89 (0.79–0.99)	6234	85%	91%
Flow T‐cell	Isolated Class I	13 (10/3)	0.80 (0.52–1.00)	8836	70%	100%
Flow T‐cell	Coexisting Class I	32 (24/8)	0.93 (0.84–1.00)	6234	92%	88%
Flow T‐cell	Coexisting Class II (control)	32 (24/8)	0.69 (0.50–0.88)	—	—	—
B. B‐cell crossmatch (combined Class I/Class II framework)						
CDC B‐cell	Isolated Class I	30 (3/27)	0.96 (0.89–1.00)	18,037	100%	96%
CDC B‐cell	Isolated Class II	23 (1/22)	NE (one positive, 9159 MFI)	—	—	—
CDC B‐cell	Coexisting Class I	50 (18/32)	0.71 (0.55–0.87)	—	—	—
CDC B‐cell	Coexisting Class II	50 (18/32)	0.67 (0.51–0.82)	—	—	—
CDC B‐cell	Coexisting overall maximum	50 (18/32)	0.76 (0.61–0.90)	18,400	67%	78%
CDC B‐cell	Coexisting summed Class I + II	50 (18/32)	0.82 (0.71–0.94)	22,133	89%	75%
CDC B‐cell	Coexisting bivariable model	50 (18/32)	0.79 (0.67–0.92)	—	—	—
CDC B‐cell	All overall maximum	103 (22/81)	0.85 (0.78–0.93)	9159	100%	60%
CDC B‐cell	All summed Class I + II	103 (22/81)	0.90 (0.85–0.96)	17,310	100%	77%
Flow B‐cell	Isolated Class I	13 (13/0)	NE (all positive, 698–22,508 MFI)	—	—	—
Flow B‐cell	Isolated Class II (exploratory)	7 (3/4)	0.83 (0.47–1.00)	825	100%	75%
Flow B‐cell	Coexisting overall maximum	32 (29/3)	0.94 (0.82–1.00)	—	—	—
Flow B‐cell	Coexisting summed Class I + II	32 (29/3)	0.97 (0.89–1.00)	—	—	—
Flow B‐cell	All overall maximum	52 (45/7)	0.90 (0.80–1.00)	2144	89%	86%

*Note:* AUC with DeLong 95% confidence interval; Youden cut‐off with its sensitivity/specificity. Because T lymphocytes express Class I only, Class II in coexisting DSA is shown as a biological control for the T‐cell crossmatch. Because B lymphocytes express both classes, class‐specific B‐cell estimates are reported only within the isolated Class I or isolated Class II subsets; in coexisting DSA, Class I and Class II are shown for completeness but without cut‐offs, alongside combined measures. Overall maximum, the single highest DSA MFI of either class. Isolated Class I, Class I DSA without Class II DSA (*n* = 30); isolated Class II, Class II DSA without Class I DSA (*n* = 23); coexisting, both classes (*n* = 50). Summed Class I + II, sum of all DSA MFI across both classes. Bivariable model, logistic regression on Class I and Class II log10 MFI (Supplementary Table S2). The paired counts are the crossmatch‐positive/negative numbers within each subset.

Abbreviations: AUC, area under the receiver‐operating‐characteristic curve; NE, not estimable.

### 3.5 B‐Cell Crossmatch Within a Combined Class I/Class II Framework

When a candidate carries both Class I and Class II DSA and the B‐cell crossmatch is positive, attribution to one class is not directly observable—a limitation others have acknowledged by excluding the B‐cell crossmatch from MFI‐correlation studies [11]. B‐cell results were therefore analysed by DSA profile (Table 3; Figure 2C–D). CDC B‐cell positivity differed across profiles: 3 of 30 (10%) with isolated Class I DSA, 1 of 23 (4%) with isolated Class II DSA and 18 of 50 (36%) with coexisting DSA (Fisher exact *p* = 0.002). In isolated Class I DSA, Class I MFI discriminated the CDC B‐cell crossmatch (AUC 0.96; 0.89–1.00), all three positives carrying at least 18,037 MFI, whereas the flow B‐cell crossmatch was positive in all 13 flow‐tested candidates at Class I MFI as low as 698, so no Class I flow threshold could be defined. In isolated Class II DSA, the single CDC B‐cell positive carried 9159 MFI, and the flow B‐cell crossmatch was positive in 3 of 7—too few events for more than an exploratory Class II threshold.

In coexisting DSA, Class I and Class II MFI were uncorrelated (Spearman *ρ* = −0.16) and each contributed independently to the CDC B‐cell crossmatch (adjusted odds ratio per 10‐fold MFI: Class I 6.65, 95% CI: 1.44–30.8; Class II 7.34, 1.64–32.9; likelihood‐ratio *p* = 0.008 and 0.003), without interaction (*p* = 0.35; Supplementary Table S2). Summed Class I + II MFI discriminated numerically better than the single strongest DSA, both in coexisting DSA (AUC 0.82 vs. 0.76) and across the cohort (0.90 vs. 0.85; paired DeLong *p* = 0.10), consistent with additive binding of both classes to the same cell. Isolated Class II antibody, mostly of low MFI, was thus accompanied by a positive flow B‐cell crossmatch only sometimes and by a positive CDC B‐cell crossmatch once, and when both classes coexist a positive B‐cell crossmatch reflects their combined load rather than either class alone.

### 3.6 Single‐Locus DSA (Exploratory)

Because several DSA usually coexisted, locus‐level data were restricted to the 42 candidates whose DSA were confined to a single locus (Figure 3; Supplementary Table S3). In this subset the CDC crossmatch was positive only with single HLA‐B DSA (T‐cell 2/7, B‐cell 1/7, all at least 14,806 MFI) and never with single HLA‐A (0/16, up to 22,508 MFI) or Class II DSA (0/19), whereas the flow B‐cell crossmatch was positive in all eight flow‐tested HLA‐A, all three HLA‐B and two of three HLA‐DRB1 candidates but in none of three with low‐MFI HLA‐DQB1 DSA. These counts are small and descriptive. Antibody‐level specificity frequencies among crossmatch‐positive candidates are provided as exploratory data only (Supplementary Table S3); when several DSA coexist, no single locus can be inferred to account for crossmatch positivity.

**FIGURE 3:**
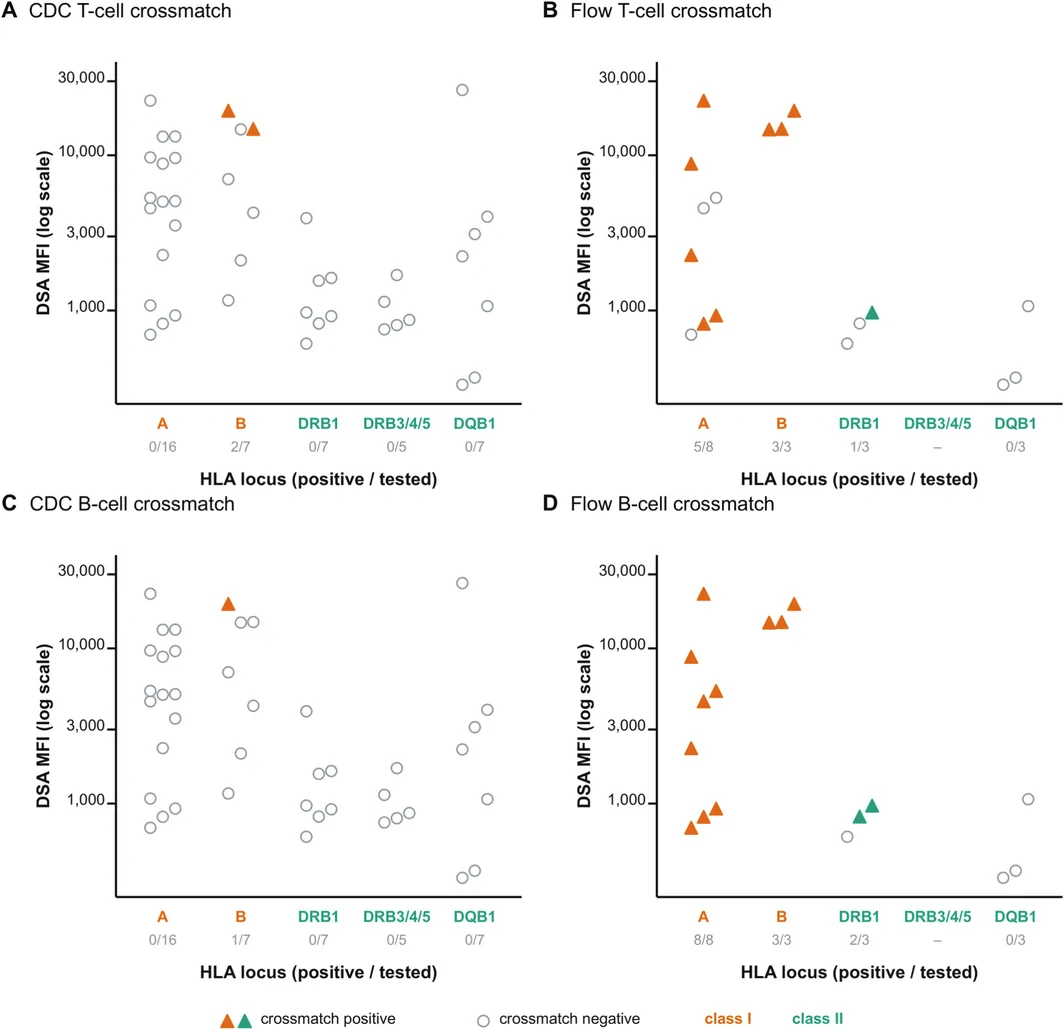
Single‐locus DSA (exploratory, patient level). Each point is one of the 42 candidates whose DSA were confined to a single HLA locus, plotted by locus and MFI (log scale); filled triangles, crossmatch positive (orange, Class I loci; teal, Class II loci); open grey circles, negative. Numbers beneath each locus give positive/tested. Candidates with DSA at more than one locus are excluded, because the locus responsible for a positive crossmatch cannot be identified when several DSA coexist.

### 3.7 Class‐Specific Decision Rules

For the T‐cell crossmatch, Class I Youden thresholds were 14,239 MFI for CDC and 6234 for flow (Figure 4A–B; Table 4). Within isolated subsets, the Class I cut‐off for the CDC B‐cell crossmatch was 18,037 MFI, while the exploratory Class II cut‐off for the flow B‐cell crossmatch was 825 MFI (sensitivity 3/3; specificity 3/4). No Class II cut‐off for CDC or Class I cut‐off for flow B‐cell crossmatches could be estimated due to insufficient positive or negative events (Figure 4C–F; Table 3).

**FIGURE 4:**
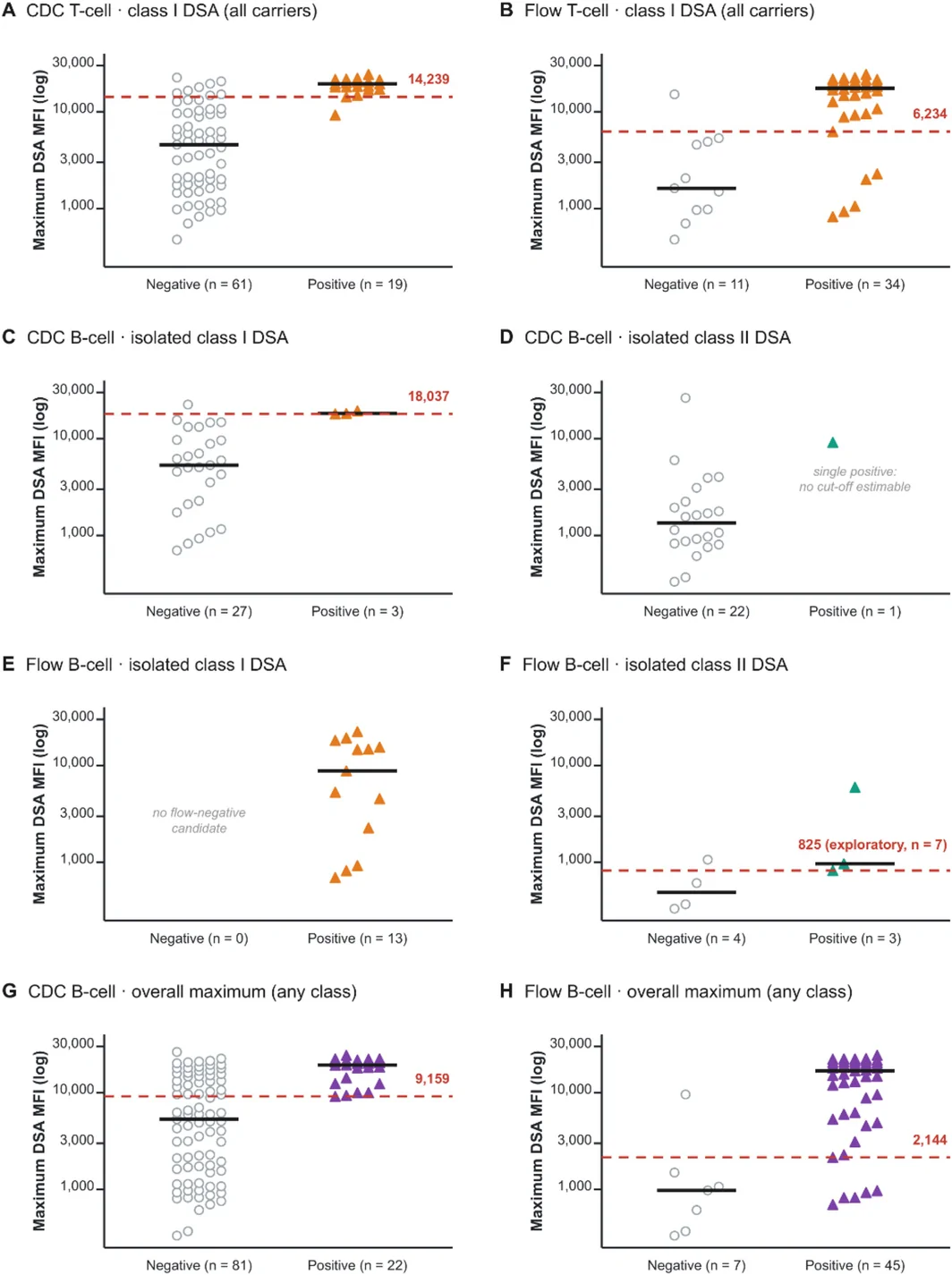
Class‐specific DSA MFI by crossmatch result. (A, B) T‐cell crossmatch against maximum Class I MFI in all candidates with Class I DSA, with the Youden cut‐off (red dashed line). (C–F) B‐cell crossmatch restricted to isolated Class I (C, E) or isolated Class II DSA (D, F), where the result can be attributed to one class, with the isolated‐subset Youden cut‐off where one could be estimated (red dashed line; Table 3): 18,037 MFI in C and an exploratory 825 MFI, from seven candidates, in F. No cut‐off could be estimated in D (a single positive) or E (no flow‐negative candidate). Filled triangles, crossmatch positive (orange, Class I; teal, Class II; purple, overall maximum); open grey circles, negative. (G, H) B‐cell crossmatch against the overall maximum MFI of either class in all candidates, with its Youden cut‐off; as a class‐independent measure it needs no attribution of the result to one class. Horizontal bars, medians.

**TABLE 4:** Decision rules and their performance.

Crossmatch	Rule	Applied to	Sens/Spec/PPV/NPV
CDC T‐cell	Class I ≥ 14,239	Class I DSA carriers (*n* = 80)	95%/87%/69%/98%
Flow T‐cell	Class I ≥ 6234	Class I DSA carriers (*n* = 45)	85%/91%/97%/71%
CDC B‐cell	Class I ≥ 16,482 or Class II ≥ 7928 (jointly optimised)	All candidates (*n* = 103)	100%/74%/51%/100%
		Isolated Class I (*n* = 30)	100%/96%/75%/100%
		Isolated Class II (*n* = 23)	100%/95%/50%/100%
		Coexisting DSA (*n* = 50)	100%/41%/49%/100%
CDC B‐cell	overall maximum ≥ 9159 (single threshold)	All candidates (*n* = 103)	100%/60%/41%/100%
		Coexisting DSA (*n* = 50)	100%/28%/44%/100%
Flow B‐cell	Class I ≥ 1615 or Class II ≥ 10,579 (jointly optimised; exploratory)	Flow‐tested (*n* = 52)	87%/100%/100%/54%
		Isolated Class I (*n* = 13)	77%/–/100%/0%
		Isolated Class II (*n* = 7)	0%/100%/–/57%
		Coexisting DSA (*n* = 32)	100%/100%/100%/100%
Flow B‐cell	overall maximum ≥ 2144 (single threshold)	Flow‐tested (*n* = 52)	89%/86%/98%/55%
		Coexisting DSA (*n* = 32)	100%/67%/97%/100%

*Note:* T‐cell rules use the Class I Youden cut‐off in candidates with Class I DSA. For the CDC and the flow B‐cell crossmatch, the either/or rule (positive if the strongest Class I DSA or the strongest Class II DSA reaches its threshold) was jointly optimised over all candidates tested and is reported overall and within each DSA profile; a candidate without DSA of a class cannot exceed that class threshold. Where several threshold pairs performed identically, the lowest is shown (CDC Class I 16,482–18,037; flow Class II 10,579–13,055 MFI). The flow B‐cell rule is exploratory (seven flow‐negatives).

Abbreviations: NPV, negative predictive value; PPV, positive predictive value.

Because isolated subsets could not supply both thresholds, a combined either/or rule was jointly optimised over all candidates (Figure 5; Table 4). For the CDC B‐cell crossmatch, the rule (Class I ≥ 16,482 or Class II ≥ 7928 MFI) yielded 100% sensitivity, 74% specificity, and 100% negative predictive value, with an optimism‐corrected Youden index of 0.69. These are parameters of a combined prediction rather than class‐specific estimates, but they were consistent with the isolated‐subset data (Table 3). Specificity was high in isolated Class I (96%) and Class II (95%) but dropped to 41% in coexisting DSA (Table 4), indicating that a rule‐positive result in coexisting DSA flags risk rather than definitive positivity. A single overall‐maximum threshold (9159 MFI; Figure 4G) demonstrated lower specificity (60%).

**FIGURE 5:**
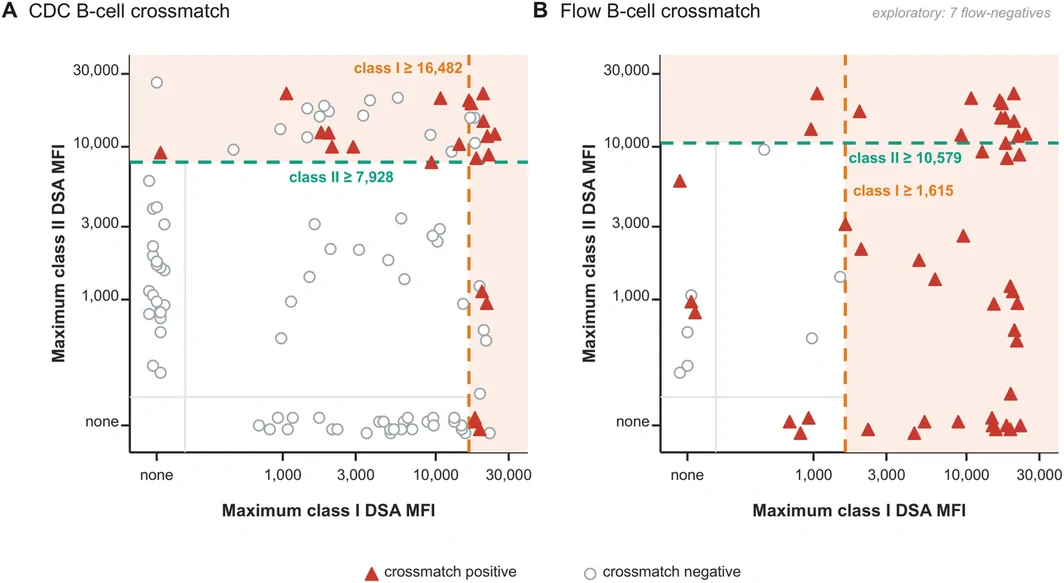
Combined Class I/Class II map for the B‐cell crossmatch. Each point is a candidate positioned by maximum Class I (x‐axis) and Class II (y‐axis) DSA MFI; candidates without DSA of a class sit in the “none” band of that axis, so isolated Class I and isolated Class II DSA lie along the bottom and left margins and coexisting DSA in the plane. Red triangles, crossmatch positive; open grey circles, negative. Both panels use the same method: the either/or rule jointly optimised over all candidates tested (orange dashed line, Class I threshold; teal dashed line, Class II threshold; shaded area, predicted positive). (A) CDC B‐cell crossmatch, Class I ≥ 16,482 or Class II ≥ 7928 MFI. (B) Flow B‐cell crossmatch, Class I ≥ 1615 or Class II ≥ 10,579 MFI; this rule is exploratory because only seven candidates were flow‐negative. Thresholds were selected by the Youden index. Prioritizing sensitivity would place the flow thresholds at Class I ≥ 698 or Class II ≥ 825 MFI (sensitivity 100%, specificity 43%), capturing the six positive candidates below the Youden thresholds; for the CDC B‐cell crossmatch (A), both criteria give the same rule.

For the flow B‐cell crossmatch, joint optimization selected Class I ≥ 1615 or Class II ≥ 10,579 MFI (87% sensitivity, 100% specificity; optimism‐corrected Youden index 0.74). This rule remains exploratory due to relying on only seven flow‐negative candidates, and it missed the three flow‐positive candidates with isolated Class II DSA. The class‐independent overall maximum yielded a Youden cut‐off of 2144 MFI (Figure 4H; Tables 3 and 4). Prioritizing sensitivity instead gave Class I ≥ 698 or Class II ≥ 825 MFI (sensitivity 100%, 45/45, 95% CI: 92%–100%; specificity 43%, 3/7, 16%–75%; negative predictive value 100%), whereas for the CDC B‐cell crossmatch the sensitivity‐first criterion selected the same rule as the Youden index, which was already fully sensitive.

### 3.8 Operating Characteristics at Fixed Clinical MFI Cut‐Offs

Operating characteristics at fixed cut‐offs of 3000, 5000, 10,000 and 15,000 MFI are given in Supplementary Table S4 and Figure 6, with B‐cell rows restricted to the overall maximum and to the isolated‐class subsets. Low cut‐offs were fully sensitive for the CDC crossmatches but missed some low‐MFI flow positives; high specificity required approximately 10,000–15,000 MFI for the CDC crossmatches but 3000–5000 for flow.

**FIGURE 6:**
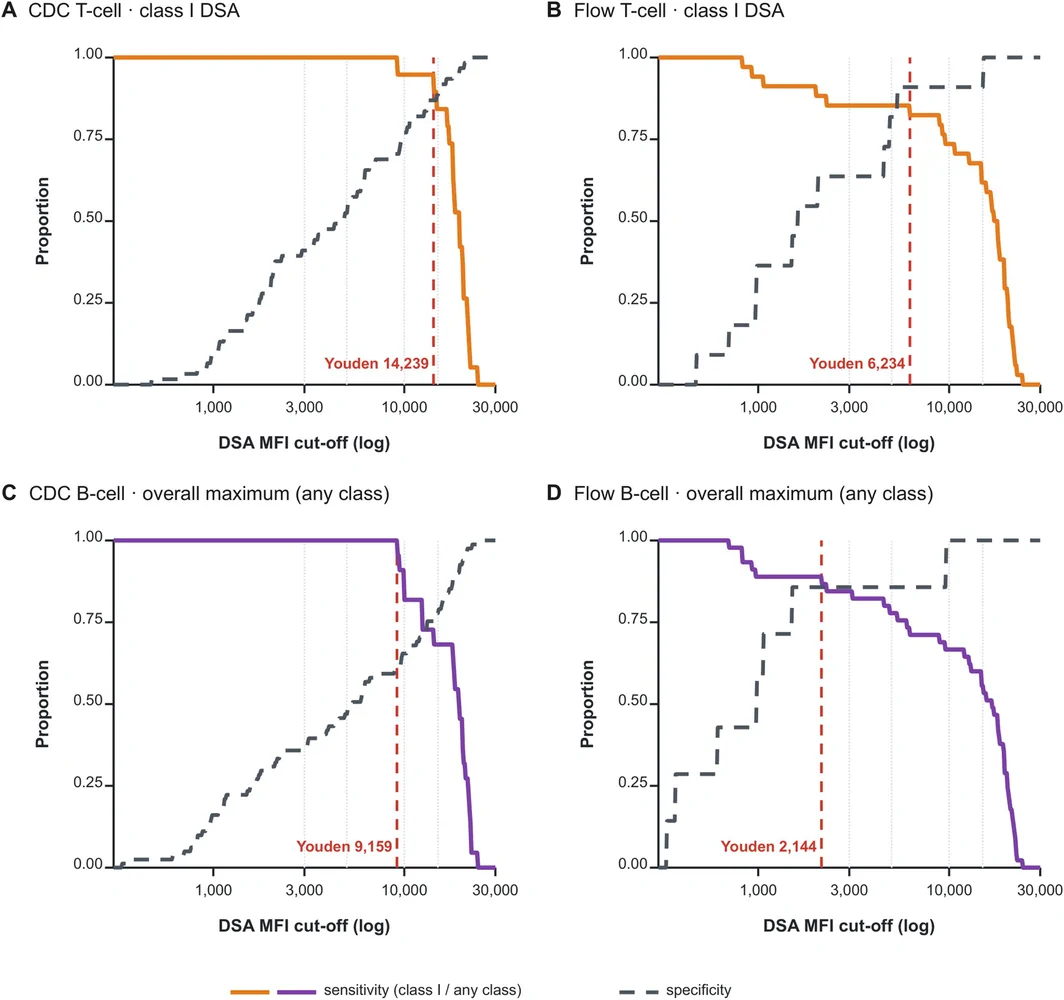
Sensitivity and specificity across MFI cut‐offs. (A, B) T‐cell crossmatch, maximum Class I MFI (sensitivity, orange); (C, D) B‐cell crossmatch, overall maximum MFI of either class (sensitivity, purple). Specificity is shown as a dark grey dashed line. Dotted lines mark the fixed cut‐offs of 3000/5000/10,000/15,000 and the red dashed line the Youden‐optimal cut‐off.

### 3.9 Robustness and Internal Validation

The findings survived standard internal checks (Supplementary Table S5). Bootstrap resampling (2000 replications) produced AUC intervals essentially identical to the DeLong estimates, and optimism‐corrected AUCs showed negligible optimism for every discriminating marker, indicating that the single‐marker models were not over‐fitted. In the pre‐specified sensitivity analysis using summed (cumulative) rather than immunodominant MFI, every conclusion was reproduced; summed thresholds were higher, as expected when several antibodies are added, while the sensitivity hierarchy, strict nesting and class‐specific pattern were unchanged.

## 4 Discussion

In 103 sensitized, DSA‐positive kidney transplant candidates with paired physical crossmatches, DSA strength, the CDC crossmatch and the flow crossmatch behaved not as three separate tests but as one ordered system. DSA MFI rose monotonically from crossmatch‐negative to flow‐positive‐only to both‐positive candidates, so that SAB, flow and CDC aligned along a single continuum of analytic sensitivity (SAB > FCXM > CDC) ordered by antibody strength (Figure 1). Flow positivity strictly contained CDC positivity, the T‐cell crossmatch followed Class I antibody alone, and the B‐cell crossmatch reflected the combined load of Class I and Class II antibody.

The value of these data lies less in any single cut‐off—several groups have reported MFI thresholds for crossmatch positivity [9, 10, 11, 12, 13, 20]—than in reconstructing the quantitative relationship among the three assays as one biologically ordered continuum within a single cohort. Framing histocompatibility testing this way shifts the question from whether a DSA is present to how far along the SAB → flow→CDC continuum it reaches, which maps directly onto the choice between virtual and physical crossmatching. Two features support this continuum. No result was CDC‐positive but flow‐negative, directly supporting the assumption behind replacing CDC with flow. And Class II antibody behaved as a biological negative control for the T‐cell crossmatch, failing to discriminate it once co‐carried Class I antibody was accounted for.

Because B lymphocytes express both HLA classes, the B‐cell crossmatch is frequently excluded from MFI‐correlation studies [11]. Stratifying the analysis by DSA profile effectively isolates the independent and combined effects of each HLA class. In isolation, Class I antibodies performed similarly on both B and T cells. Conversely, isolated Class II antibodies—predominantly of low MFI in this cohort—rarely yielded a positive CDC B‐cell crossmatch (1/23 candidates). This aligns with prior reports of lower B‐cell flow crossmatch sensitivity for Class I‐negative/Class II‐positive DSA, where reliable positivity typically requires an MFI exceeding 5000 [15]. Although that previous study utilized a SAB‐defined virtual crossmatch as the reference for a rapid flow crossmatch and restricted its B‐cell analysis to isolated Class II DSA [15], the present study expands upon this framework. Specifically, this analysis incorporates the CDC crossmatch, evaluates both cell‐based crossmatches as outcomes predicted by continuous MFI, and directly models candidates with coexisting DSA—a subgroup comprising half of the current cohort and accounting for 18 of the 22 CDC B‐cell positive results. When both classes coexisted, they contributed independently without evidence of statistical interaction.

Indeed, evaluating a single global MFI cut‐off (the overall maximum) yielded substantially lower specificity across the cohort (60% vs. 74%), demonstrating that a universal threshold fails to account for the distinct biological limits of each class. To address this, a jointly optimized “either/or” rule was derived. This joint rule successfully captured 100% of CDC B‐cell positive cases across every DSA profile (100% NPV). However, even with this optimized approach, specificity within the coexisting DSA subset remained modest (41%), although still superior to that of the overall maximum (28%). This indicates that while a rule‐negative result reliably predicts compatibility, a rule‐positive virtual crossmatch in coexisting DSA should prompt a physical crossmatch to guide pre‐ or post‐transplant management—such as antibody removal therapy or induction selection—rather than definitively precluding transplantation.

For the flow B‐cell crossmatch, published thresholds typically cluster between 2000 and 4000 MFI [9, 27, 28]. Notably, the class‐independent overall maximum threshold derived in the present cohort (2144 MFI) falls squarely at the lower boundary of this historical range. However, when decomposed by HLA class, a distinct divergence emerges: the jointly optimized Class I threshold (1615 MFI) aligns more closely with this lower bound, whereas the Class II threshold (10,579 MFI) lies substantially above it. The elevated Class II estimate reflects the disproportionate influence of a single flow‐negative candidate, while the sensitivity‐first thresholds (698 and 825 MFI) rest on a few low‐MFI positive results. These low‐MFI positives may represent pronase‐enhanced B‐cell sensitivity [29] or unrecognized antibodies directed against untyped HLA loci. Consequently, these specific flow B‐cell cut‐offs remain strictly exploratory and should be used cautiously beyond this laboratory.

These distinctions matter as programs, including the national deceased‐donor programme in Thailand, move toward virtual crossmatching, and as centres that have eliminated the physical crossmatch report safe outcomes with shorter cold‐ischemia time [7, 30, 31]. Safe adoption requires reading a SAB report quantitatively and by HLA class, recognizing antibody in the flow‐positive but sub‐CDC range that the older assay would have missed. The same numbers extend beyond crossmatch safety, flagging candidates whose antibody strength may warrant more intensive immunosuppression and defining a quantitative target—reduction below the relevant threshold—for desensitization and its monitoring [6, 32].

Several limitations apply. Flow crossmatch was available in only 52 of 103 candidates, with few flow‐negatives, so flow‐specificity intervals are wide, the nesting is partly conditional on referral for flow, and the flow B‐cell rule is exploratory: its Class II threshold was set by a single flow‐negative candidate, it missed the three flow‐positive candidates with isolated Class II DSA, and the isolated Class II estimate (825 MFI) rests on seven candidates. The isolated‐class subsets that make class attribution unambiguous were small, particularly for Class II, in which CDC B‐cell positivity was a single event. Raw, non‐baseline‐adjusted MFI from untreated serum was used without EDTA treatment or dilution studies; therefore prozone inhibition [8, 33] may have underestimated the strongest antibodies, and bead saturation compresses the upper range. Reactivity to denatured HLA on single‐antigen beads, which does not bind intact cells [34], may have inflated MFI in some crossmatch‐negative candidates, and kit‐lot and instrument effects, which shift MFI between runs and laboratories [35], could not be standardised across the study period. Donor typing at antigen level for HLA‐A, ‐B, ‐DRB1, ‐DRB3/4/5 and ‐DQB1 meant that antibodies to HLA‐C, ‐DQA1 and ‐DPB1, and allele‐specific DSA, could not be assigned. Serial dilution and bead‐by‐bead normalisation are not yet standard [36, 37]; the absolute cut‐offs are therefore laboratory‐specific and the Youden values may be optimistic, whereas the fixed‐cut‐off table is more transportable. Most fundamentally, this is a test–test study without clinical endpoints, so the thresholds describe assay behaviour rather than rejection risk.

These findings complement outcome studies that a test–test design cannot replace. In living‐donor recipients, combining flow and solid‐phase assays with HLA eplet matching best stratified rejection risk [38], and in deceased‐donor recipients a positive flow crossmatch and DSA number—but not MFI alone—independently predicted antibody‐mediated rejection [39]. The class‐specific thresholds derived here are therefore best positioned not as a replacement for cellular testing but as one quantitative input alongside complement‐binding assays [40], molecular (eplet) mismatch, and emerging computational risk models—with prospective validation against rejection and graft survival the essential next step.

In sensitized kidney transplant candidates, SAB, flow and CDC crossmatching form a single, HLA‐class‐ordered continuum of antibody detection in which flow positivity nests CDC positivity and each physical crossmatch turns positive at a reproducible MFI that is class‐specific for the T‐cell crossmatch and reflects combined Class I and Class II load for the B‐cell crossmatch. Rather than proposing another universal cut‐off, these data offer a biologically validated, class‐ and assay‐specific framework for anticipating a physical crossmatch from a SAB report and for deciding when a physical crossmatch adds information beyond the virtual crossmatch. This framework supplies part of the quantitative bridge toward safe MFI‐based virtual crossmatching.

## Author Contributions

N.C. planned the study, collected data, performed the laboratory analysis, and wrote the first manuscript draft. S.O. and P.C. performed laboratory analysis. N.H. reviewed and edited the manuscript. S.U. designed the study, performed statistical analysis, reviewed and edited the manuscript, and supervised the project.

## Funding

The authors have nothing to report.

## Disclosure

The authors have nothing to report.

## Ethics Statement

The study was approved by the Research Ethics Committee, National Blood Centre, Thai Red Cross Society (NBC 4/2569), and conducted in accordance with the Declaration of Helsinki. Because it used existing, de‐identified laboratory data, individual informed consent was waived. The study adhered to the Declaration of Istanbul on Organ Trafficking and Transplant Tourism; no organs were procured from executed prisoners or from any paid or coerced donor, and all donations were voluntary. All data were anonymized before analysis.

## Conflicts of Interest

The authors declare no conflicts of interest.

## Supporting information


**Supplementary Table S1:** DSA profile of candidates with a CDC T‐cell‐positive/B‐cell‐negative crossmatch.
**Supplementary Table S2:** Multivariable logistic regression of physical‐crossmatch positivity on donor‐specific antibody strength.
**Supplementary Table S3:** DSA by HLA locus.
**Supplementary Table S4:** Operating characteristics at fixed clinical MFI cut‐offs.
**Supplementary Table S5:** Internal validation of each discrimination model in the revised framework.
**Supplementary Figure S1:** Discrimination (AUC, 95% CI, DeLong) of DSA MFI for each crossmatch.

## Data Availability

The data that support the findings of this study are available from the corresponding author upon reasonable request.
