# Supplementary material for: From Single‐Antigen Beads to the Physical Crossmatch: A Class‐Specific MFI Framework for Virtual Crossmatching in Kidney Transplantation
Source: J Clin Lab Anal. 2026 Nov 13:e70400. Online ahead of print. doi: 10.1002/jcla.70400 (PMC13768760; doi:10.1002/jcla.70400)
Supplement: Supplementary file 1 [file JCLA-9999-e70400-s001.docx]

**Supplementary Table S1. DSA profile of candidates with a CDC T-cell-positive/B-cell-negative crossmatch.** Maximum DSA MFI per class and flow crossmatch results. All six T-cell-positive/B-cell-negative candidates carried strong class I DSA and were positive in both lineages by flow, indicating a false-negative B-cell CDC reading. Pos, positive.

| **Candidate** | **DSA profile** | **Max class I MFI** | **Max class II MFI** | **Flow T-cell** | **Flow B-cell** |
| --- | --- | --- | --- | --- | --- |
| 1 | Isolated class I | 14,806 | none | Pos | Pos |
| 2 | Coexisting | 21,349 | 539 | Pos | Pos |
| 3 | Coexisting | 16,811 | 15,507 | Pos | Pos |
| 4 | Coexisting | 19,361 | 1,225 | Pos | Pos |
| 5 | Coexisting | 18,037 | 10,579 | Pos | Pos |
| 6 | Coexisting | 9,234 | 11,980 | Pos | Pos |

**Supplementary Table S2. Multivariable logistic regression of physical-crossmatch positivity on donor-specific antibody strength.** Maximum (immunodominant) class I and class II donor-specific antibody (DSA) mean fluorescence intensity (MFI), each on the log10 scale, were entered simultaneously to test whether one HLA class predicts a positive crossmatch independently of the other. Odds ratios (OR) are per 10-fold (one log10-unit) increase in MFI; unadjusted ORs are single-predictor models fitted on the same candidates. Analyses were restricted to candidates carrying both classes of DSA, since an adjusted class effect is defined only when both are present (B-cell model n = 50, 18 CDC B-cell positive; T-cell models n = 50 [CDC] and 32 [flow]).

| **Predictor (per 10-fold increase in maximum MFI)** | **Unadjusted OR (95% CI)** | **p** | **Adjusted OR (95% CI)** | **p** |
| --- | --- | --- | --- | --- |
| CDC B-cell — Maximum class I DSA MFI | 4.01 (1.06–15.21) | 0.041 | 6.65 (1.44–30.82) | 0.015 |
| CDC B-cell — Maximum class II DSA MFI | 5.08 (1.25–20.74) | 0.023 | 7.34 (1.64–32.92) | 0.009 |
| CDC T-cell — maximum class II DSA MFI (adj. class I)† | 1.28 (0.41–4.04) | 0.67 | 3.38 (0.73–15.7) | 0.12 |
| Flow T-cell — maximum class II DSA MFI (adj. class I)† | 2.89 (0.72–11.6) | 0.14 | 23.8 (0.38–1485) | 0.13 |

For the B-cell crossmatch, class II DSA contributed independently of class I: adding class II to a model already containing class I was significant (likelihood-ratio χ²[1] = 8.92, p = 0.003), and class I and class II MFI were uncorrelated (Spearman ρ = −0.16), indicating distinct information. The same model applied to the T-cell crossmatch is a biological specificity control: because T lymphocytes do not express HLA class II, class II should not predict the T-cell crossmatch, and after adjustment for class I it did not (CDC adjusted OR 3.38, p = 0.12; flow 23.8, p = 0.13; both non-significant unadjusted). Consistently, isolated class II DSA (no class I; n = 23) never produced a positive CDC T-cell crossmatch (0/23), and 24 of 26 (92%) flow T-cell-positive class II carriers co-carried class I DSA — identifying the modest flow signal as co-sensitization, not class II recognition by T cells. Estimator: the CDC B-cell model used standard maximum likelihood; the T-cell models (†) used Firth penalized logistic regression because the dominant class I effect caused quasi-complete separation, which inflates maximum-likelihood odds ratios; the flow B-cell model, separated by only 3 flow-negative candidates, is summarized by its likelihood-ratio test (χ²[1] = 8.09, p = 0.005) without stable odds ratios. Wide confidence intervals reflect subgroup size. Additional checks in the coexisting-DSA subset (n = 50): class I added to class II (likelihood-ratio χ²[1] = 7.06, p = 0.008); there was no class I × class II interaction (χ²[1] = 0.86, p = 0.35); and the bivariable model discriminated the CDC B-cell crossmatch with AUC 0.79 (95% CI 0.67–0.92; optimism-corrected 0.77).

†Firth penalized logistic regression, adjusted for maximum class I DSA MFI.

OR, odds ratio; CI, confidence interval; DSA, donor-specific antibody; MFI, mean fluorescence intensity; CDC, complement-dependent cytotoxicity.

**Supplementary Table S3. DSA by HLA locus.** (A) Candidates whose DSA were confined to a single HLA locus (patient level; n = 42): DSA MFI and crossmatch positivity (positive/tested) by locus. (B) Exploratory antibody-level frequency of DSA specificities among crossmatch-positive candidates. Panel B counts antibody occurrences, several of which come from the same candidate, so they are not independent observations; because several DSA usually coexist, no specificity in panel B can be inferred to account for crossmatch positivity. In panel B, N = antibody occurrences at each locus/allele and % = within that locus for the group (locus total = 100%); bold rows are locus totals. Denominators: CDC T+ n = 19, CDC B+ n = 22, FCXM T+ n = 36, FCXM B+ n = 45.

| **Locus / specificity** | **CDC T-cell** | | **CDC B-cell** | | **Flow T-cell** | | **Flow B-cell** | |
| --- | --- | --- | --- | --- | --- | --- | --- | --- |
| **A. Candidates with DSA at a single HLA locus (patient level, n = 42): crossmatch positive / tested** | | | | | | | | |
| HLA-A (n = 16) MFI 5,051 (698–22,508) | 0/16 | | 0/16 | | 5/8 | | 8/8 | |
| HLA-B (n = 7) MFI 7,020 (1,162–19,386) | 2/7 | | 1/7 | | 3/3 | | 3/3 | |
| HLA-DRB1 (n = 7) MFI 970 (612–3,934) | 0/7 | | 0/7 | | 1/3 | | 2/3 | |
| HLA-DRB3/4/5 (n = 5) MFI 869 (756–1,693) | 0/5 | | 0/5 | | not tested | | not tested | |
| HLA-DQB1 (n = 7) MFI 2,235 (332–26,366) | 0/7 | | 0/7 | | 0/3 | | 0/3 | |
| **B. DSA specificities among crossmatch-positive candidates (antibody level, exploratory): N and % within locus** | | | | | | | | |
|  | **N** | **%** | **N** | **%** | **N** | **%** | **N** | **%** |
| **HLA-A** | **13** | **100.00** | **17** | **100.00** | **27** | **100.00** | **33** | **100.00** |
| A1 | 2 | 15.38 | 2 | 11.76 | 4 | 14.81 | 4 | 12.12 |
| A2 | 3 | 23.08 | 3 | 17.65 | 7 | 25.93 | 8 | 24.24 |
| A3 | 2 | 15.38 | 1 | 5.88 | 2 | 7.41 | 2 | 6.06 |
| A11 | 1 | 7.69 |  |  | 3 | 11.11 | 6 | 18.18 |
| A24 | 1 | 7.69 | 4 | 23.53 | 6 | 22.22 | 8 | 24.24 |
| A25 | 1 | 7.69 | 1 | 5.88 | 1 | 3.70 | 1 | 3.03 |
| A29 | 1 | 7.69 |  |  | 1 | 3.70 | 1 | 3.03 |
| A30 |  |  | 2 | 11.76 |  |  |  |  |
| A31 |  |  | 2 | 11.76 |  |  |  |  |
| A33 | 1 | 7.69 | 1 | 5.88 | 2 | 7.41 | 2 | 6.06 |
| A66 | 1 | 7.69 | 1 | 5.88 | 1 | 3.70 | 1 | 3.03 |
| **HLA-B** | **28** | **100.00** | **26** | **100.00** | **42** | **100.00** | **46** | **100.00** |
| B7 | 2 | 7.14 | 2 | 7.69 | 2 | 4.76 | 2 | 4.35 |
| B8 | 3 | 10.71 | 3 | 11.54 | 6 | 14.29 | 6 | 13.04 |
| B13 |  |  |  |  | 1 | 2.38 | 1 | 2.17 |
| B18 | 1 | 3.57 | 4 | 15.38 | 3 | 7.14 | 3 | 6.52 |
| B27 | 1 | 3.57 | 1 | 3.85 | 1 | 2.38 | 1 | 2.17 |
| B35 | 2 | 7.14 | 2 | 7.69 | 5 | 11.90 | 5 | 10.87 |
| B38 | 1 | 3.57 | 1 | 3.85 | 2 | 4.76 | 2 | 4.35 |
| B39 | 2 | 7.14 | 1 | 3.85 | 2 | 4.76 | 2 | 4.35 |
| B44 | 1 | 3.57 | 1 | 3.85 | 1 | 2.38 | 2 | 4.35 |
| B46 |  |  |  |  | 1 | 2.38 | 1 | 2.17 |
| B47 | 1 | 3.57 |  |  | 1 | 2.38 | 1 | 2.17 |
| B50 |  |  |  |  | 1 | 2.38 | 1 | 2.17 |
| B51 | 2 | 7.14 | 2 | 7.69 | 1 | 2.38 | 1 | 2.17 |
| B52 | 1 | 3.57 | 1 | 3.85 | 2 | 4.76 | 3 | 6.52 |
| B56 | 3 | 10.71 | 1 | 3.85 | 2 | 4.76 | 2 | 4.35 |
| B57 | 2 | 7.14 | 2 | 7.69 | 4 | 9.52 | 4 | 8.70 |
| B58 | 2 | 7.14 | 2 | 7.69 | 2 | 4.76 | 2 | 4.35 |
| B60 | 1 | 3.57 |  |  | 1 | 2.38 | 2 | 4.35 |
| B61 |  |  |  |  | 1 | 2.38 | 1 | 2.17 |
| B62 | 3 | 10.71 | 2 | 7.69 | 3 | 7.14 | 4 | 8.70 |
| B75 |  |  | 1 | 3.85 |  |  |  |  |
| **HLA-DRB1** | **12** | **100.00** | **16** | **100.00** | **21** | **100.00** | **23** | **100.00** |
| DR3 | 2 | 16.67 | 2 | 12.50 | 2 | 9.52 | 2 | 8.70 |
| DR4 | 4 | 33.33 | 1 | 6.25 | 7 | 33.33 | 7 | 30.43 |
| DR7 | 1 | 8.33 | 2 | 12.50 | 3 | 14.29 | 3 | 13.04 |
| DR8 |  |  |  |  |  |  | 1 | 4.35 |
| DR11 | 1 | 8.33 | 2 | 12.50 | 3 | 14.29 | 3 | 13.04 |
| DR12 | 3 | 25.00 | 5 | 31.25 | 3 | 14.29 | 3 | 13.04 |
| DR14 |  |  | 2 | 12.50 | 1 | 4.76 | 2 | 8.70 |
| DR15 | 1 | 8.33 | 2 | 12.50 | 2 | 9.52 | 2 | 8.70 |
| **HLA-DRB3/4/5** | **1** | **100.00** | **6** | **100.00** | **2** | **100.00** | **4** | **100.00** |
| DR51 |  |  | 1 | 16.67 |  |  |  |  |
| DR52 |  |  | 4 | 66.67 | 1 | 50.00 | 3 | 75.00 |
| DR53 | 1 | 100.00 | 1 | 16.67 | 1 | 50.00 | 1 | 25.00 |
| **HLA-DQB1** | **16** | **100.00** | **16** | **100.00** | **26** | **100.00** | **28** | **100.00** |
| DQ2 | 4 | 25.00 | 5 | 31.25 | 7 | 26.92 | 8 | 28.57 |
| DQ5 | 2 | 12.50 | 2 | 12.50 | 3 | 11.54 | 3 | 10.71 |
| DQ6 | 1 | 6.25 | 1 | 6.25 | 1 | 3.85 | 1 | 3.57 |
| DQ7 | 1 | 6.25 | 3 | 18.75 | 5 | 19.23 | 6 | 21.43 |
| DQ8 | 8 | 50.00 | 5 | 31.25 | 10 | 38.46 | 10 | 35.71 |

**Supplementary Table S4. Operating characteristics at fixed clinical MFI cut-offs.** T-cell crossmatch: maximum class I DSA MFI in all class I carriers and overall maximum MFI. B-cell crossmatch, by DSA profile: class I MFI in isolated class I DSA, class II MFI in isolated class II DSA, and overall maximum MFI in coexisting DSA and in all candidates; class-specific B-cell rows in coexisting DSA are omitted because a positive B-cell crossmatch cannot be attributed to one class. Sensitivity, specificity, positive (PPV) and negative predictive value (NPV) at 3,000, 5,000, 10,000 and 15,000 MFI; – , not estimable.

| **Crossmatch** | **Subset · measure** | **n (pos)** | **MFI cut-off** | **Sens** | **Spec** | **PPV** | **NPV** |
| --- | --- | --- | --- | --- | --- | --- | --- |
| CDC T-cell | class I (all with class I DSA) | 80 (19) | 3,000 | 100% | 41% | 35% | 100% |
| CDC T-cell | class I (all with class I DSA) | 80 (19) | 5,000 | 100% | 52% | 40% | 100% |
| CDC T-cell | class I (all with class I DSA) | 80 (19) | 10,000 | 95% | 77% | 56% | 98% |
| CDC T-cell | class I (all with class I DSA) | 80 (19) | 15,000 | 84% | 89% | 70% | 95% |
| CDC T-cell | overall maximum (all) | 103 (19) | 3,000 | 100% | 35% | 26% | 100% |
| CDC T-cell | overall maximum (all) | 103 (19) | 5,000 | 100% | 45% | 29% | 100% |
| CDC T-cell | overall maximum (all) | 103 (19) | 10,000 | 100% | 65% | 40% | 100% |
| CDC T-cell | overall maximum (all) | 103 (19) | 15,000 | 84% | 80% | 48% | 96% |
| Flow T-cell | class I (all with class I DSA) | 45 (34) | 3,000 | 85% | 64% | 88% | 58% |
| Flow T-cell | class I (all with class I DSA) | 45 (34) | 5,000 | 85% | 82% | 94% | 64% |
| Flow T-cell | class I (all with class I DSA) | 45 (34) | 10,000 | 74% | 91% | 96% | 53% |
| Flow T-cell | class I (all with class I DSA) | 45 (34) | 15,000 | 62% | 91% | 95% | 43% |
| Flow T-cell | overall maximum (all) | 52 (36) | 3,000 | 89% | 56% | 82% | 69% |
| Flow T-cell | overall maximum (all) | 52 (36) | 5,000 | 89% | 75% | 89% | 75% |
| Flow T-cell | overall maximum (all) | 52 (36) | 10,000 | 78% | 88% | 93% | 64% |
| Flow T-cell | overall maximum (all) | 52 (36) | 15,000 | 67% | 94% | 96% | 56% |
| CDC B-cell | class I (isolated class I DSA) | 30 (3) | 3,000 | 100% | 30% | 14% | 100% |
| CDC B-cell | class I (isolated class I DSA) | 30 (3) | 5,000 | 100% | 41% | 16% | 100% |
| CDC B-cell | class I (isolated class I DSA) | 30 (3) | 10,000 | 100% | 78% | 33% | 100% |
| CDC B-cell | class I (isolated class I DSA) | 30 (3) | 15,000 | 100% | 93% | 60% | 100% |
| CDC B-cell | class II (isolated class II DSA) | 23 (1) | 3,000 | 100% | 77% | 17% | 100% |
| CDC B-cell | class II (isolated class II DSA) | 23 (1) | 5,000 | 100% | 91% | 33% | 100% |
| CDC B-cell | class II (isolated class II DSA) | 23 (1) | 10,000 | 0% | 95% | 0% | 95% |
| CDC B-cell | class II (isolated class II DSA) | 23 (1) | 15,000 | 0% | 95% | 0% | 95% |
| CDC B-cell | overall maximum (coexisting DSA) | 50 (18) | 3,000 | 100% | 12% | 39% | 100% |
| CDC B-cell | overall maximum (coexisting DSA) | 50 (18) | 5,000 | 100% | 22% | 42% | 100% |
| CDC B-cell | overall maximum (coexisting DSA) | 50 (18) | 10,000 | 94% | 34% | 45% | 92% |
| CDC B-cell | overall maximum (coexisting DSA) | 50 (18) | 15,000 | 67% | 53% | 44% | 74% |
| CDC B-cell | overall maximum (all) | 103 (22) | 3,000 | 100% | 36% | 30% | 100% |
| CDC B-cell | overall maximum (all) | 103 (22) | 5,000 | 100% | 47% | 34% | 100% |
| CDC B-cell | overall maximum (all) | 103 (22) | 10,000 | 91% | 65% | 42% | 96% |
| CDC B-cell | overall maximum (all) | 103 (22) | 15,000 | 68% | 78% | 45% | 90% |
| Flow B-cell | class I (isolated class I DSA) | 13 (13) | 3,000 | 69% | – | 100% | 0% |
| Flow B-cell | class I (isolated class I DSA) | 13 (13) | 5,000 | 62% | – | 100% | 0% |
| Flow B-cell | class I (isolated class I DSA) | 13 (13) | 10,000 | 46% | – | 100% | 0% |
| Flow B-cell | class I (isolated class I DSA) | 13 (13) | 15,000 | 31% | – | 100% | 0% |
| Flow B-cell | class II (isolated class II DSA) | 7 (3) | 3,000 | 33% | 100% | 100% | 67% |
| Flow B-cell | class II (isolated class II DSA) | 7 (3) | 5,000 | 33% | 100% | 100% | 67% |
| Flow B-cell | class II (isolated class II DSA) | 7 (3) | 10,000 | 0% | 100% | – | 57% |
| Flow B-cell | class II (isolated class II DSA) | 7 (3) | 15,000 | 0% | 100% | – | 57% |
| Flow B-cell | overall maximum (coexisting DSA) | 32 (29) | 3,000 | 97% | 67% | 97% | 67% |
| Flow B-cell | overall maximum (coexisting DSA) | 32 (29) | 5,000 | 90% | 67% | 96% | 40% |
| Flow B-cell | overall maximum (coexisting DSA) | 32 (29) | 10,000 | 83% | 100% | 100% | 38% |
| Flow B-cell | overall maximum (coexisting DSA) | 32 (29) | 15,000 | 72% | 100% | 100% | 27% |
| Flow B-cell | overall maximum (all) | 52 (45) | 3,000 | 84% | 86% | 97% | 46% |
| Flow B-cell | overall maximum (all) | 52 (45) | 5,000 | 78% | 86% | 97% | 38% |
| Flow B-cell | overall maximum (all) | 52 (45) | 10,000 | 67% | 100% | 100% | 32% |
| Flow B-cell | overall maximum (all) | 52 (45) | 15,000 | 56% | 100% | 100% | 26% |

**Supplementary Table S5. Internal validation of each discrimination model in the revised framework.** Apparent AUC = fit on all data; bootstrap 95% CI from 2,000 stratified resamples; optimism-corrected AUC from the Harrell bootstrap. The jointly optimised either/or rules (CDC and flow B-cell) are validated by their Youden index, re-deriving both thresholds in each of 1,000 bootstrap samples (Stata). Subsets without crossmatch-negative candidates, or with a single positive, are not shown.

| **Crossmatch** | **Subset · measure** | **Apparent AUC** | **Bootstrap 95% CI** | **Optimism-corrected AUC** |
| --- | --- | --- | --- | --- |
| CDC T-cell | All with class I DSA · class I | 0.94 | 0.89–0.99 | 0.94 |
| CDC T-cell | Isolated class I · class I | 0.95 | 0.87–1.00 | 0.95 |
| CDC T-cell | Coexisting · class I | 0.94 | 0.87–0.99 | 0.94 |
| CDC T-cell | Coexisting · class II (control) | 0.50 | 0.33–0.67 | 0.44 |
| Flow T-cell | All with class I DSA · class I | 0.89 | 0.77–0.97 | 0.88 |
| Flow T-cell | Isolated class I · class I | 0.80 | 0.50–1.00 | 0.77 |
| Flow T-cell | Coexisting · class I | 0.93 | 0.83–1.00 | 0.93 |
| Flow T-cell | Coexisting · class II (control) | 0.69 | 0.49–0.86 | 0.67 |
| CDC B-cell | Isolated class I · class I | 0.96 | 0.89–1.00 | 0.97 |
| CDC B-cell | Coexisting · overall maximum | 0.76 | 0.61–0.89 | 0.76 |
| CDC B-cell | Coexisting · summed class I + II | 0.82 | 0.70–0.92 | 0.83 |
| CDC B-cell | All · overall maximum | 0.85 | 0.77–0.92 | 0.85 |
| CDC B-cell | All · summed class I + II | 0.90 | 0.84–0.95 | 0.90 |
| Flow B-cell | Isolated class II · class II (exploratory) | 0.83 | 0.50–1.00 | 0.84 |
| Flow B-cell | Coexisting · overall maximum | 0.94 | 0.79–1.00 | 0.95 |
| Flow B-cell | Coexisting · summed class I + II | 0.97 | 0.86–1.00 | 0.97 |
| Flow B-cell | All · overall maximum | 0.90 | 0.79–0.98 | 0.90 |
| CDC B-cell | Coexisting · bivariable model | 0.79 | 0.65–0.90 | 0.77 |
| CDC B-cell | All · either/or rule (Youden index) | 0.74 | – | 0.69 |
| Flow B-cell | Flow-tested · either/or rule (Youden index; exploratory) | 0.87 | – | 0.74 |

**Supplementary Figure S1. Discrimination (AUC, 95% CI, DeLong) of DSA MFI for each crossmatch.** T-cell crossmatch by class I DSA, overall and by DSA profile, with class II in coexisting DSA as the biological control (open circles); B-cell crossmatch within the combined class I/class II framework (isolated-class subsets, coexisting DSA and all candidates). Colours: orange, class I; teal, class II; blue, summed class I + II; purple, overall maximum.
